# Altered Dopamine Signaling in Naturally Occurring Maternal Neglect

**DOI:** 10.1371/journal.pone.0001974

**Published:** 2008-04-09

**Authors:** Stephen C. Gammie, Michelle N. Edelmann, Caleigh Mandel-Brehm, Kimberly L. D'Anna, Anthony P. Auger, Sharon A. Stevenson

**Affiliations:** 1 Department of Zoology, University of Wisconsin-Madison, Madison, Wisconsin, United States of America; 2 Neuroscience Training Program, University of Wisconsin-Madison, Madison, Wisconsin, United States of America; 3 Department of Psychology, University of Wisconsin-Madison, Madison, Wisconsin, United States of America; Minnesota State University Mankato, United States of America

## Abstract

**Background:**

Child neglect is the most common form of child maltreatment, yet the biological basis of maternal neglect is poorly understood and a rodent model is lacking.

**Methodology/Principal Findings:**

The current study characterizes a population of mice (MaD1) which naturally exhibit maternal neglect (little or no care of offspring) at an average rate of 17% per generation. We identified a set of risk factors that can predict future neglect of offspring, including decreased self-grooming and elevated activity. At the time of neglect, neglectful mothers swam significantly more in a forced swim test relative to nurturing mothers. Cross-fostered offspring raised by neglectful mothers in turn exhibit increased expression of risk factors for maternal neglect and decreased maternal care as adults, suggestive of possible epigenetic contributions to neglect. Unexpectedly, offspring from neglectful mothers elicited maternal neglect from cross-fostered nurturing mothers, suggesting that factors regulating neglect are not solely within the mother. To identify a neurological pathway underlying maternal neglect, we examined brain activity in neglectful and nurturing mice. c-Fos expression was significantly elevated in neglectful relative to nurturing mothers in the CNS, particularly within dopamine associated areas, such as the zona incerta (ZI), ventral tegmental area (VTA), and nucleus accumbens. Phosphorylated tyrosine hydroxylase (a marker for dopamine production) was significantly elevated in ZI and higher in VTA (although not significantly) in neglectful mice. Tyrosine hydroxylase levels were unaltered, suggesting a dysregulation of dopamine activity rather than cell number. Phosphorylation of DARPP-32, a marker for dopamine D1-like receptor activation, was elevated within nucleus accumbens and caudate-putamen in neglectful versus nurturing dams.

**Conclusions/Significance:**

These findings suggest that atypical dopamine activity within the maternal brain, especially within regions involved in reward, is involved in naturally occurring neglect and that MaD1 mice are a useful model for understanding the basis of naturally occurring neglect.

## Introduction

Child neglect is the most common form of child maltreatment and is highly debilitating. Although often considered together, child neglect and abuse are separable processes in humans and other primates [1], [2] with neglect accounting for greater than one-half of national maltreatment reports [3]. Further, child neglect is thought to be responsible for more than one-third of maltreatment related deaths each year [3]. When not lethal, child neglect can be devastating by adversely affecting childhood development [4] and elevating risks for depression and deficits in cognitive, academic, and social performance [5], [6]. Exposure to neglect negatively affects adult outcomes and is associated with higher neglect of offspring in the next generation [7]–[9]. Despite advances in understanding the behavioral profiles of neglect, the biological basis of neglect is poorly understood and interventions are only slowly being developed [2]. It has been suggested that child neglect involves abnormalities in parental appetitive motivation (lack of hedonic reward from children) rather than an inability to perform a particular parental behavior [10].

Dopamine is considered to be a key player in reward-related behaviors [11] and because maternal neglect may involve a lack of reward from offspring, dysregulation of dopamine is a candidate mechanism for neglect. Previous work in rodents suggests a role for dopamine in maternal neglect. For example, maternal care is disrupted with dopamine receptor antagonists [12]–[15] or with lesions of nucleus accumbens and ventral tegmental area (VTA) [16]–[19], key regions in the mesolimbic dopamine anticipation-reward pathway. Although a number of rodent studies have identified pharmacological manipulations or lesions that decrease maternal care [10], disrupting maternal care does not necessarily shed light on the normal basis of maternal neglect. Further, very few studies in rodents have addressed how to mitigate maternal neglect. Some studies have explored the basis of low levels of licking of pups [20], [21], but these studies do not examine maternal neglect per se. To our knowledge, we are the first to identify the regular appearance of maternal neglect within an otherwise normal population and then try to understand the basis of that neglect.

In these studies, we describe a unique mouse model with consistent rates of naturally occurring maternal neglect within an otherwise nurturing population. The mice producing high levels of maternal neglect were originally one of four lines of mice selected for high wheel-running behavior from outbred (hsd:ICR) mice [22]. In a previous study, no overt changes in maternal care were found to occur with selection for high wheel-running [23]. In 2001, we observed that one of the four high wheel-running lines of mice had very high levels of defense of offspring (maternal aggression) [24] and then maintained that line our lab for over 17 generations using selection for high maternal defense. We refer to these mice as high maternal defense 1 (MaD1) mice. In this study, we also employ a cross-fostering approach to determine how maternal neglect affects offspring performance as adults. We examine brain activity to test the hypothesis that a dysregulation of dopamine signaling underlies this naturally occurring form of neglect. A key goal of these studies is to develop a model for understanding the basis of naturally occurring neglect and how neglect affects offspring.

## Results

### Maternal neglect in MaD1 mice

**Figure 1:**
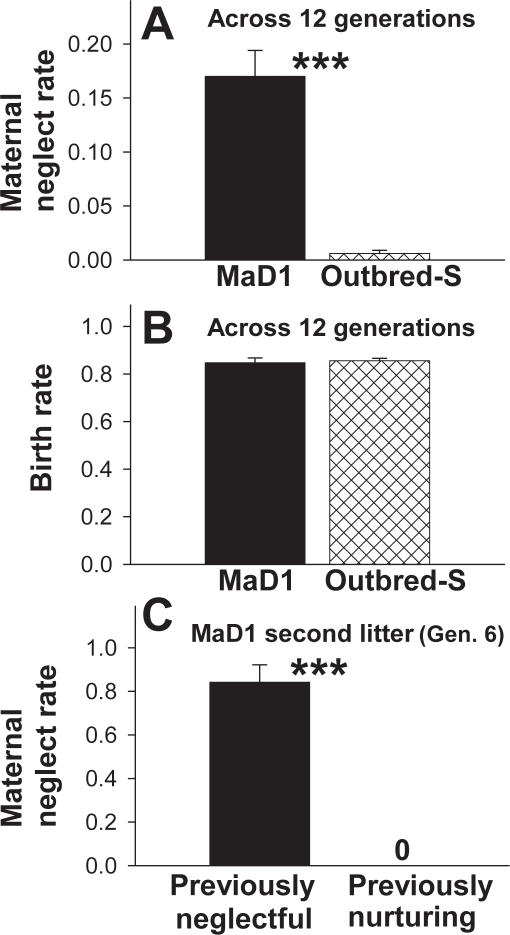
Maternal neglect rate in MaD1 mice. Maternal neglect rate (neglect leads to death of all pups in a litter) was significantly higher in MaD1 relative to Outbred-S mice when examined over 12 generations (A). Birth rate (number of male-female pairings relative to number of litters born) is almost identical between MaD1 and Outbred-S mice when examined over 12 generations (B). When raising a second litter (examined in Generation 6), previously neglectful mice exhibited significantly higher levels of maternal neglect relative to previously nurturing MaD1 mice (C). Bars represent means±SE. ***  = p<0.001.

When examined over 12 generations (∼80 lactating females evaluated per generation per group), MaD1 mice [25] had a significantly higher average neglect rate (∼17%) relative to Outbred-S mice (<1%) (Fig. 1A, H(1,23) = 17.56, p<0.001; ANOVA on Ranks). Maternal neglect rate each generation was determined by examining the number of live litters on postpartum Day 5 relative to the number of live litters born. In contrast, birth rates (litters born relative to male-female matings) over these 12 generations were almost identical between MaD1 and Outbred-S mice (Fig. 1B, F (1,23) = 0.08, p = 0.778). Neglect is a relatively stable trait across litters from an individual. In Generation 6, 84% of previously neglectful females neglected their second litter, whereas no previously nurturing mice neglected (Fig. 1C, H (1,28) = 18.13, p<0.001; ANOVA on Ranks).

In most generations, maternal behaviors were not recorded, but when they were recorded, they were based on undisturbed observations. In Generation 16, first time MaD1 mothers were examined three times per day to provide a profile of maternal neglect. During this generation, 9 mothers were neglectful. Within about 80% of the neglected litters, milk was apparent within the pups (Table 1). On average, the last day nursing and the first appearance of a dead pup occurred on about 1.5 days (0 = postpartum Day 0) (Table 1). The average day for all pups to be found dead was 3 (Table 1). Infanticide or cannibalization was not common. In only 1 of 9 cases were more than 2 pups from a given litter found to be partially eaten and in most cases no cannibalization was observed. Rather, the most common form of pup death was from neglect. In Generation 17, on postpartum Day 0 for mice on their second litter, mice that would eventually neglect their pups (relative to those that would nurture) showed a significantly longer time to retrieve their first pup (122.7±44.7 versus 11.2±2.2 sec) (H (1,31) = 11.8, p<0.001, ANOVA on Ranks) and fifth pup (208.3±37.3 versus 52.1±5.4 sec) (H (1,31) = 12.4, p<0.001, ANOVA on Ranks). This result suggests that pup retrieval may be a quick and reliable measure for quickly identifying neglectful mothers.

**Table 1:** Descriptive measures of litters that would eventually be neglected in Generation 16.

Descriptive Measure	Average	Standard Error
Proportion of litters with milk bands in pups	0.78	±0.14
Last day of nursing	1.67	±0.50
Appearance of 1^st^ dead pup (days)	1.67	±0.57
All pups dead (days)	2.89	±0.56

### Possible risk factors for maternal neglect in MaD1 mice

**Figure 2:**
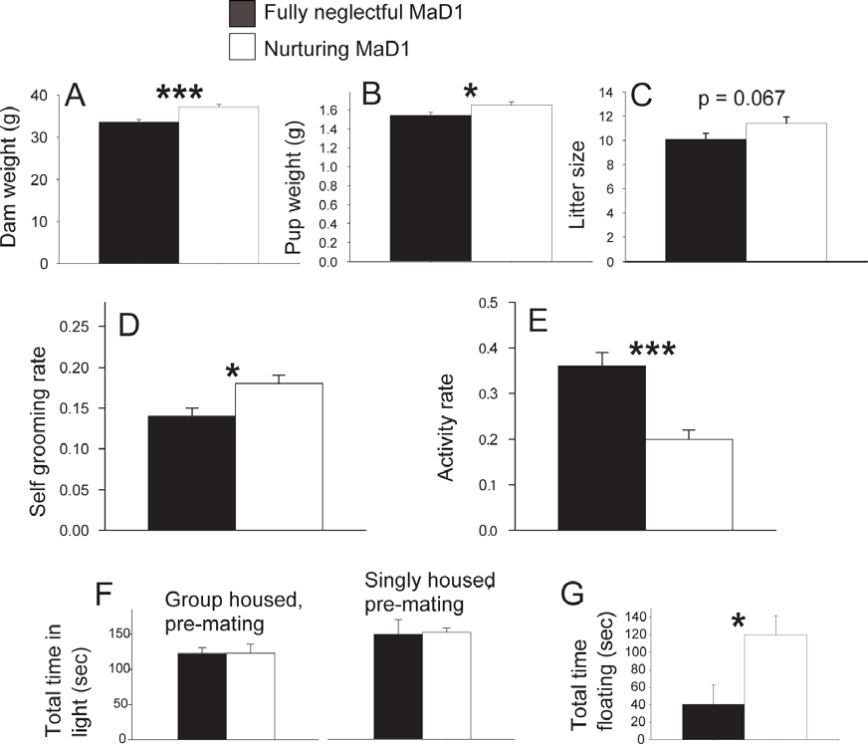
Possible risk factors for maternal neglect in MaD1 mice across multiple generations. When examined for the first litter in on postpartum Day 0, neglectful MaD1 mice weighed significantly less relative to their nurturing sisters (A) and average pup weight was significantly lower for pups born to neglectful mothers (B). Litter size was smaller for neglectful mice, but levels did not reach significance(C). Prior to mating, decreases in self-grooming (D) and increases in activity (E) were seen in neglectful relative to nurturing mice (D). For A–C, data from Generations (G) 12 and 15 were combined and for D and E, data from G6, G12, and G15 were combined. Time in light in a light/dark box test, did not differ between mice that would become either neglectful or nurturing when examined in G15 (F). Mice were first examined for anxiety at age 50 when they were group housed and then again after being singly housed for one week. In G17, neglectful mice spent significantly less time floating in the forced swim test relative to nurturing mice (G). Bars represent means±SE. *  = p<0.05; ***  = p<0.001.

We examined general traits across different generations determine whether any of these varied with neglect. Using a 2-way ANOVA analysis that included generation number as a variable, we found that dam weight measured on postpartum Day 0 was significantly lower in neglectful mice relative to nurturing mice (Fig. 2A) (F (1,74) = 11.8, p<0.001). Further, pups born to neglectful mice weighed significantly less relative to pups born to nurturing females (Fig. 2B) (F (1,75) = 5.6, p = 0.02). Litter size was lower in neglectful mice, but levels did not reach significance (Fig. 2C) (F(1,61) = 3.4, p = 0.067). Self-grooming measured before mating was significantly lower in neglectful relative to nurturing females (Fig. 2D) (F (1,157) = 5.7, p = 0.018). Neglectful females had higher activity levels when examined before mating (Fig. 2E) (F (1,157) = 15.4, p<0.001). No differences were observed in terms of total time in the light portion of the light/dark box test in mice first examined at age 50 while being group housed (F (1,69) = 0.02, p = 0.871) or again after being singly housed for one week (F (1,22) = 0.00, p = 0.991) (Fig. 2F). None of the other light/dark box measures differed between groups (data not shown). In Generation 17, at the time of first neglect (∼Day 1), neglectful mice were found to spend significantly more time swimming (F (1,17) = 6.3, p = 0.023) and significantly less time floating (F (1,17) = 6.5, p = 0.021) (Fig. 2G) than nurturing mice. Further, the number of fecal boli produced during the swim test was significantly lower in neglectful (0.11±0.11) versus nurturing (2.22±0.52) mice (H (1,17) = 10.4, p<0.001, ANOVA on Ranks).

### Mid-cross-fostering results: maternal behaviors and effect of offspring on maternal neglect

**Figure 3:**
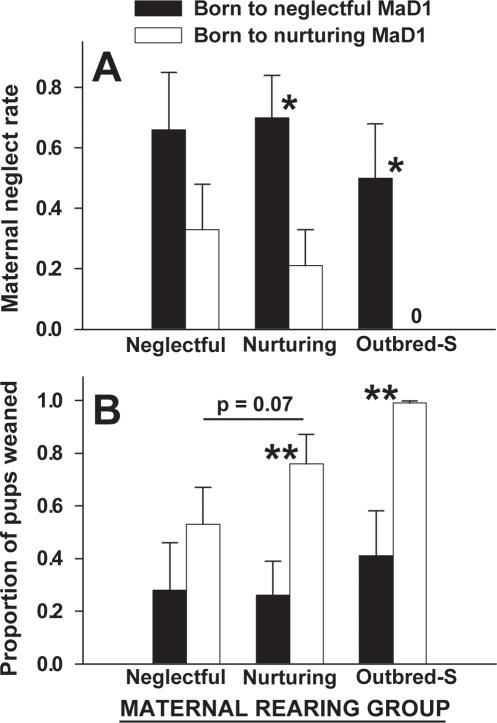
Pups born to previously neglectful mice elicit decreases in maternal care. When raised by either previously nurturing MaD1 mice or Outbred-S mice, pups that were born to previously neglectful MaD1 mothers received significantly higher levels of maternal neglect relative to pups born to previously nurturing MaD1 mice (A). Similarly, the proportion of pups weaned was significantly lower for pups born to previously neglectful MaD1 mothers when raised by either previously nurturing MaD1 mothers or Outbred-S mothers (B). Previously neglectful (relative to nurturing) MaD1 mice weaned a lower proportion of pups when the pups were from nurturing mothers, but the differences did not reach significance (p = 0.071). Bars represent means±SE. *  = p<0.05; **  = p<0.01.

Unexpectedly, pups born to previously neglectful (relative to nurturing) MaD1 mothers were significantly more likely to receive maternal neglect (F (1,50) = 11.3, p = 0.002); 2-way ANOVA). This effect was clearly seen when neglectful pups were being raised by either previously nurturing MaD1 (H (1,22) = 5.4, p = 0.02; ANOVA on Ranks) or Outbred-S mice (H (1,17) = 6.6, p = 0.01; ANOVA on Ranks) (Fig. 3A). A difference in neglect of pups was not seen in previously neglectful MaD1 mothers, but this may be due to the 30% neglect rate of previously nurturing pups (Fig. 3A). The proportion of pups weaned was significantly lower for pups born to previously neglectful (relative to nurturing) MaD1 mothers (F (1,57) = 20.3, p<0.001; 2-way ANOVA). This effect was again seen when neglectful pups were raised by either previously nurturing MaD1 (H (1,22) = 6.7, p = 0.009; ANOVA on Ranks) or Outbred-S mice (H (1,17) = 9.4, p = 0.002; ANOVA on Ranks) (Fig. 3B).

When comparing proportion of nurturing MaD1 pups weaned between neglectful and nurturing MaD1 mothers, heightened neglect was seen in the neglectful mothers but this did not reach significance (p = 0.07) (Fig. 3B).

Although a main goal of the cross-fostering study was to examine how offspring would fare as adults when given different rearing environments, during the pup rearing phase we conducted one maternal behavior observation for 1 hour on postpartum Day 3. No significant differences were observed between previously neglectful and nurturing MaD1 mothers, although a consistent trend towards lower maternal care was seen among neglectful mothers (data not shown). Negative effects of neglectful mothers on offspring were seen when offspring were adults (see below). So it is possible that with prolonged maternal care observations, significant differences between groups could be identified.

### Effect of neglectful MaD1 mothers on offspring behavior as adults

**Figure 4:**
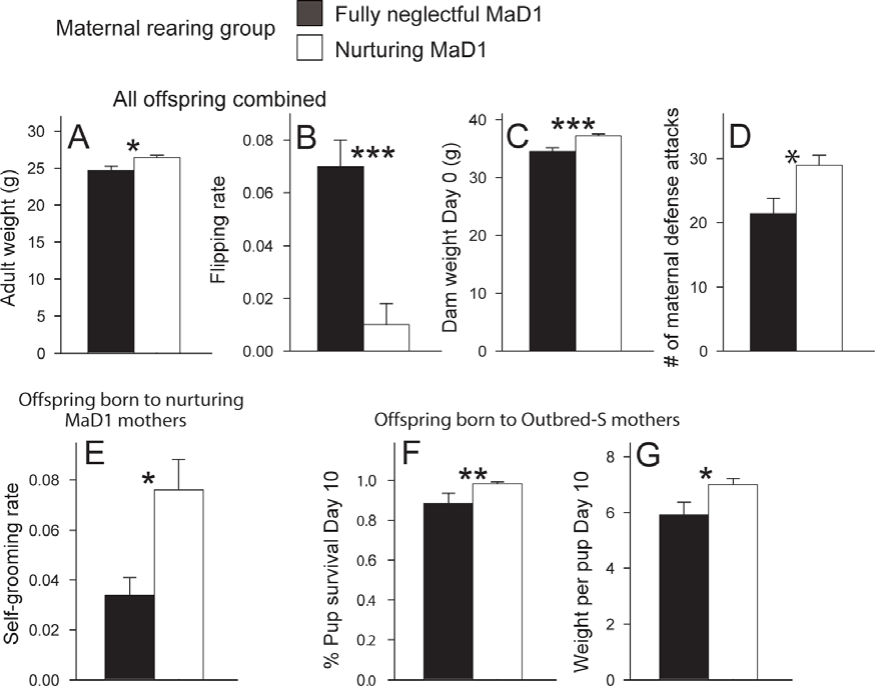
Cross-fostered offspring raised by previously neglectful (relative to nurturing) MaD1 mothers show deficits as adults. When results from all offspring were combined, previously neglectful MaD1 mothers negatively impacted offspring adult performance in terms of body weight pre-mating (A), flipping rate pre-mating (B), body weight on postpartum Day 0 (C), and number of maternal defense attacks (D). Additionally, when just offspring from previously nurturing MaD1 mothers were examined, they exhibited significantly lower levels self-grooming pre-mating when raised by a neglectful mother (E). When just offspring from Outbred-S mothers were examined as adults, deficits in terms of proportion of pups that survive to postpartum Day 10 (a marker of maternal neglect) (F), and the average weight of pups on postpartum Day 10 (G) were observed when these mice were raised by neglectful MaD1 mothers. Bars represent means±SE. *  = p<0.05; **  = p<0.01.

Previously neglectful (relative to nurturing) MaD1 mothers imparted deficits on offspring they were raising when offspring were examined as adults. When using a 2-way ANOVA to examine the overall effect of neglectful versus nurturing mothers when raising pups from all three pup groups, adult weight of female offspring raised by neglectful mothers was significantly lower relative to those raised by nurturing dams (Fig. 4A) (F(1,120) = 7.5, p = 0.007). Flipping rate pre-mating, a measure of hyperactivity, was also higher in offspring that had been raised by neglectful relative to nurturing females (Fig. 4B) (F(1,120) = 10.8, p = 0.001, ANOVA on Ranks). Weight of offspring at postpartum Day 0 (when they gave birth) was significantly lower when offspring were raised by neglectful relative to nurturing mice (Fig. 4C) (F(1,119) = 14.1, p<0.001). Offspring raised by neglectful mothers were significantly less likely to protect their own pups in terms of expression of number of attacks against an intruder (Fig. 4D) (F(1,102) = 6.8, p = 0.01). No other measures differed between offspring raised by neglectful relative to nurturing mothers using a 2-way ANOVA analysis.

Interestingly, planned comparisons revealed negative impacts of being reared by neglectful MaD1 mothers that were specific to offspring genotype. For example, decreased self-grooming rate as adults was observed if the offspring were from previously nurturing MaD1 mothers (Fig. 4E) (F (1,37) = 4.2, p = 0.047). When the offspring being raised were from Outbred-S mothers a decreased percentage of pup survival on Day 10 (a marker of neglect) (Fig. 4F) (F (1,37) = 7.9, p = 0.005; ANOVA on Ranks), and decreased average weight per pup on Day 10 (a marker of neglect) (Fig. 4G) (F (1,44) = 5.3, p = 0.025) was found if these mice had been reared by previously neglectful mothers.

### c-Fos, pTH, TH, and pDARPP-32 expression in neglectful MaD1 mice

**Figure 5:**
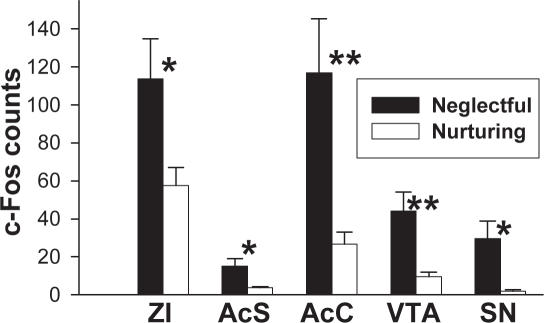
Altered c-Fos expression with maternal neglect. Heightened c-Fos expression in dopamine releasing and responding regions in neglectful (N = 8) relative to nurturing (N = 9) mice. Significantly higher levels of c-Fos are found in ZI, VTA, and substantia nigra (SN), all of which are involved in dopamine production. Both nucleus accumbens shell (AcS) and core (AcC), which respond to dopamine signaling, also show increased c-Fos in neglectful mice. Other regions examined for c-Fos are shown in Table 1. Bars represent means±SE. *  = p<0.05; **  = p<0.01.

**Figure 6:**
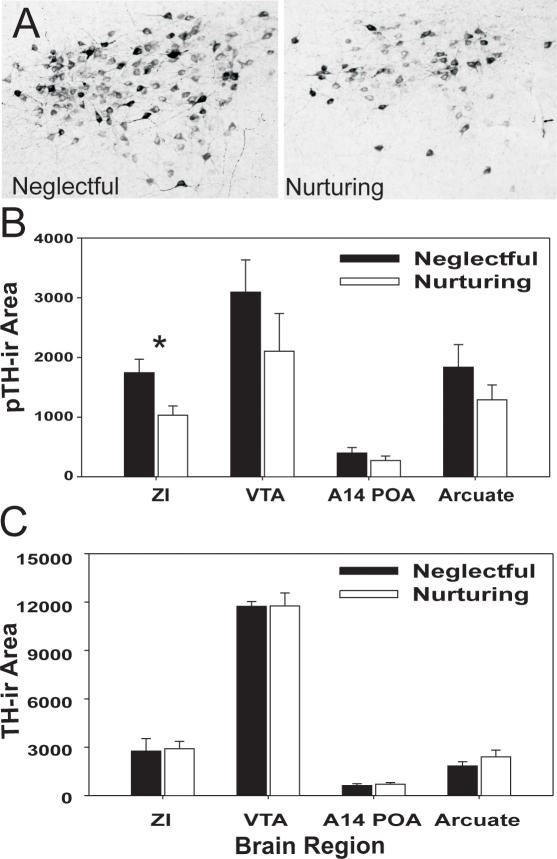
Altered pTH expression with maternal neglect. Example of heightened pTH immunoreactivity in ZI in neglectful relative to nurturing mice is shown in (A). Significant elevations of pTH-ir area are found in ZI, but not other dopamine producing regions, in neglectful mice (N = 7) in comparison to nurturing dams (8) (B). TH-ir area does not differ between neglectful (N = 8) and nurturing (N = 8) mice. ZI = zona incerta, VTA = ventral tegmental area, A14 POA = A14 region of preoptic area. Bars represent means±SE. *  = p<0.05.

**Figure 7:**
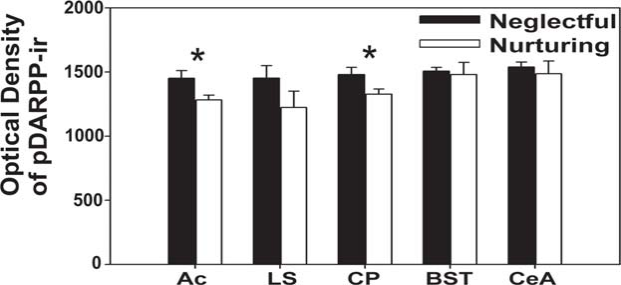
Altered pDARPP-32 expression with maternal neglect. Previously neglectful dams (N = 5) have significantly darker optical density of pDARPP-32 expression in Ac and CP when compared to previously nurturing dams (N = 6). Ac = nucleus accumbens, LS = lateral septum, CP = caudate-putamen, BST = bed nucleus of stria terminalis, dorsal, CeA = central amygdala. Bars represent means±SE. *  = p<0.05.

Neglectful females show region specific differences in c-Fos, phosphorylated tyrosine hydroxylase (pTH), and phosphorylated DARPP-32 (pDARPP-32) expression compared to their nurturing counterparts. In particular, c-Fos expression was significantly higher in neglectful (N  = 8) versus nurturing mice (N = 9) in dopamine releasing and responding regions (Fig. 5, p<0.05), as well as additional regions (Table 2, p<0.05). Neglectful females (N = 7) express greater serine 40 (ser40) phosphorylated TH immunoreactive (pTH-ir) cell and fiber area compared to nurturing females (N = 8) in the zona incerta (ZI) (Fig. 6A, B, p<0.05). There were no differences in pTH-ir within the ventral tegmental area, A14 region of the preoptic area, or the arcuate (Fig. 6B, p>0.05). Furthermore, no difference in TH-ir area was observed within any of these regions, including ZI (Fig. 6C, p>0.05, N = 8 each). The activity of D1-like dopamine receptor-associated processes was investigated by examining the phosphorylation of DARPP-32 in dams with their second litter. While there was no difference in the number of pDARPP-32-ir cells within the lateral septum, caudate- putamen (CP), bed nucleus of the stria terminalis dorsal, central amygdala, or the nucleus accumbens (Ac), previously neglectful dams (N = 5) had more intense cellular optical density of pDARPP-32 immunoreactivity than previously nurturing (N = 6) dams within the Ac and CP (Fig. 7, p<0.05). The greater cellular optical density of pDARPP-32 staining with the Ac and CP suggests a greater level of dopamine D1-like receptor activity within these regions. The regional specificity of greater optical density of pDARPP-32 immunoreactivity occurring within the Ac of neglecting dams was replicated in a smaller study examining dams that were neglecting their first litter (N = 4 neglectful, N = 3 nurturing, p<0.05, data not shown).

**Table 2:** Mean (±SE) number of c-Fos positive cells in additional brain regions in neglectful and nurturing mice.

Brain Region	Neglectful	Nurturing	p-value
LSV	31.3±6.0	6.6±2.6	p<0.001
CG	119.2±26.3	7.7±2.1	p<0.001 #
MeA	102.3±24.5	16.8±5.6	p = 0.004 #
CeA	57.0±25.6	12.0±2.7	p = 0.030 #
LH	23.8±7.0	5.6±2.3	p = 0.012 #
Pir	265.0±62.1	24.6±5.7	p = 0.005 #
PVN	36.0±7.8	12.7±3.4	p = 0.013
cPAG	32.1±5.0	13.5±2.3	p = 0.004
MPOM	77.5±18.6	38.6±15.9	p = 0.083 #
MPA	45.5±18.5	34.2±4.5	p = 0.665
BNSTv	19.6±3.1	8.0±2.2	p = 0.008
AHA	22.1±6.0	9.5±2.3	p = 0.136 #
SCN	71.5±18.9	90.0±18.4	p = 0.248 #
cPAG1	31.0±9.5	17.7±3.7	p = 0.210 #

See Fig. 6 for c-Fos in dopamine releasing and responding regions.

Abbreviations: LSV = lateral septum ventral; CG = cingulate cortex; MeA = medial amygdala; CeA = central amygdala; LH = lateral hypothalamus; Pir = piriform cortex; PVN = paraventricular nucleus; cPAG = caudal periaqueductal gray; cPAG1 = more caudal aspect of cPAG; MPOM = medial preoptic nucleus; MPA = medial preoptic area; AHA = anterior hypothalamic area; SCN = suprachiasmatic nucleus. #- data non-normal, analyzed with ANOVA on Ranks.

## Discussion

We suggest MaD1 mice can be utilized as a powerful tool for examining the biological basis of naturally occurring neglect. This line of mice can consistently produce a subset of neglectful mothers (∼17% each generation) (Fig. 1A). Interestingly, in a given group of sisters, five may be outstanding mothers in all respects, but one will give birth and then show limited or no maternal response such that all pups within a litter die in a few days. Besides the higher percentage of neglect in MaD1 mice, this line shows many of the same maternal traits as another line of mice (Outbred-S) that are also derived from outbred hsd:ICR mice via selection. For example, both MaD1 and Outbred-S mice exhibit high maternal defense (protection of offspring) against a male intruder. Therefore, high maternal defense does not appear linked to neglect of offspring. Birth rate is also similar in both MaD1 and Outbred-S mice (Fig. 1B), indicating a fertility related issue is not likely the basis for neglect.

Interestingly, this study indicated dysregulated dopamine signaling in neglectful MaD1 mice. This may prove very pertinent because in humans, abnormalities in parental appetitive motivation (lack of hedonic reward from children) rather than an inability to perform a particular parental behavior is implicated in some cases of child neglect [10]. Importantly, even when previously neglectful mice do raise litters (via cross-fostering), they negatively affect offspring's fitness as adults as indicated by decreased maternal performance and increased expression of risk factors for maternal neglect. This impact of mother on offspring's long-term behavior may also be relevant to the epigenetic transmission of neglect seen in humans.

### Possible risk factors for maternal neglect

By examining behavior both prior to mating, during pregnancy, and at the time of birth, we were able to determine predictors, or risk factors, that were associated with maternal neglect (Fig. 2). Because none of the risk factors (body weight, dam weight, pup weight, litter size, self-grooming, activity) are all or none and maternal neglect ends in complete abandonment, it is not likely that any of the risk factors are the cause of neglect, but rather reveal underlying neural or physiological differences that increase the likelihood of neglect. A lower birth weight has been found to correlate with elevated neglect in humans in some studies [26], but not in others [27].

Low self-grooming (Fig. 2D) is interesting because grooming is considered to be a predictor of health in humans and mice. Activity (a measure that included any in cage locomotor movement, such as running or cage climbing) was also significantly higher in mice that would become neglectful (Fig. 2E). Activity is an intriguing risk factor because in humans hyperactivity, often in the form of attention deficit hyperactivity disorder (ADHD), may involve dysregulation of dopamine signaling [28] and adult ADHD is associated with poor monitoring of child behavior [29]. Anxiety was not found to be a risk factor for neglect as measured using the light/dark box (Fig. 2F). The finding of elevated swimming in neglectful mice at the time of neglect (postpartum Day 1) (Fig. 2G) was interesting because decreased swimming has been linked to possible depressive behavior in rodents [30]–[32], whereas elevated swimming has been linked to mania [33]. Mania in humans can involve a lack of modulation of brain activity in nucleus accumbens with reward [34] and it would be interesting if a key contributor to neglect was an inability of neglectful mice to modulate dopamine levels in nucleus accumbens in response to pups.

### Pups born to neglectful mothers induce neglect

It is unclear why pups born to previously neglectful mice were more likely to be neglected by two of the three maternal groups in the cross-fostering study (Fig. 3). In some instances, previously nurturing mice actively scattered the pups from previously neglectful MaD1 mice and did not give them the chance to nurse or develop, suggesting a negative cue or lack of a positive cue is provided by these pups. Although not commonly examined in maternal neglect studies, an understanding of why certain offspring are more likely to elicit neglect is an important component for a comprehensive understanding of the biology of neglect. Most studies of neglect examine the mother; however, the current data indicate that the pup's behavior can itself elicit neglect.

### Previously neglectful mothers adversely affect offspring trajectory

Together, we see a deleterious environmental effect of being reared by previously neglectful mothers that manifests itself in terms of lower adult body weight, heightened flipping, decreased dam weight, and decreased number of maternal defense attacks (Fig. 4). Further, when focusing on Outbred-S offspring, it was found that being raised by previously neglectful mothers decreased maternal care in terms of the number of live pups and pup weight on Day 10 (Figs. 4F–G), suggesting a possible epigenetic transmission of neglect across generations. It is not too surprising that no differences were observed in maternal care during cross-fostering given that maternal behavior was only monitored once for one hour. Repeated and detailed monitoring of maternal care during a future cross-fostering may reveal interesting insights into how maternal behavioral differences lead to altered adult profiles.

How the rearing environment provided by previously neglectful MaD1 mothers negatively affects offspring performance is not known, but recent work suggests epigenetic contributions to elevated activity in rats [35]. Also, low levels of licking by a dam leads to a heightened stress reactivity of offspring and DNA methylation is thought to underlie the epigenetic effect [36]. Although this latter study, as well as others, examines variations in maternal care, these studies do not examine maternal neglect per se, especially the extreme levels that alter mortality rates of offspring. In humans, receiving neglect during childhood increases the likelihood of being neglectful as an adult [7]–[9] and it will be interesting to understand how rearing by previously neglectful MaD1 mice triggers decreases in maternal care when cross-fostered offspring are adults.

### Dopamine dysregulation in maternal neglectful

There are region specific differences in the brain of neglectful versus nurturing mice. We found altered c-Fos activity (an indirect marker for neuronal activity) in many brain regions that are important for dopamine signaling, maternal behavior, or both. Elevated c-Fos in nucleus accumbens shell region (AcS) and core region (AcC) is interesting because these are targets of dopamine neurotransmission from VTA [11] and, in rats, activation of nucleus accumbens as revealed by fMRI has recently been associated with suckling by pups that may occur via VTA [37]. Nucleus accumbens is part of the maternal care circuit [38], [39] and dopamine release is increased in nucleus accumbens in rats in response to pups [40], [41]. Dopamine release within AcS is involved in pair bonding in voles [42], which may have correlates with mother-infant bonding. The elevated c-Fos activity in VTA, ZI, and substantia nigra, regions enriched with dopamine producing enzymes, suggests alterations in some aspects of dopamine signaling. Heightened c-Fos activity in bed nucleus of stria terminalis, ventral, a component of a core maternal behavior circuitry [10], and LS (Table 1), a region involved in maternal defense (protection of offspring) [43], [44], could reflect altered dopamine release into these regions from ZI [45]. The patterning of c-Fos activity in dopamine releasing and responding regions encouraged us to examine levels of TH, the rate limiting enzyme of dopamine.

We observed a key difference in the activational state of TH in neglectful mice. The phosphorylation of TH is involved in the short term regulation of this enzyme [46] and although TH can be phosphorylated at Ser8, Ser19, Ser31, and Ser40, the phosphorylation of TH at Ser40 (examined here) is correlated with the greatest increase in dopamine synthesis and release [47]–[50]. In this study, ser40 pTH activity, but not TH expression, was elevated in neglectful mice in ZI (Fig. 6). ZI is a region well-known for normal dopamine synthesis and sends projections to maternal behavior regions, including medial preoptic region [45]. Although TH is also involved in norepinephrine (NE) synthesis, ZI contains a high number of dopaminergic neurons [51], [52] and the dopamine beta hydroxylase enzyme necessary for NE production has little or no expression in ZI, so pTH activity there is thought to reflect dopamine synthesis.

As we find no difference in overall levels of TH, these findings suggest a difference in the activity of dopamine neurons rather than in neuronal number within neglectful MaD1 mice. Elevated pTH activity in ZI does not simply reflect underlying differences in enzyme expression and instead, might be due to neglectful mice having a heightened input to ZI. In future work it will be important to examine in neglectful mice possible differences in dopamine receptors and inputs to dopamine systems.

Another useful approach to examine whether dopamine activity is heightened in neglectful mice is to examine the postsynaptic phosphorylational state of DARPP-32. Phosphorylation of DARPP-32 is increased by D1-like, but not D2-like, dopamine receptors and is specifically phosphorylated on threonine34 (Thr34) in response to dopamine acting through a cAMP-dependent protein kinase (PKA) [53]–[55]. Although not all DARPP-32 expressing neurons contain D1-like dopamine receptors, DARPP-32 is present in practically all D1-like receptor-containing neurons [56], and is generally regarded as a useful marker to determine where increased D1-like receptor activity is occurring within the brain. Interestingly, DARPP-32 has also been implicated in reward processes, as suggested by DARPP-32 KO mice that lack rewarding effects of ethanol as illustrated by a conditioned place preference test [57]. In agreement with higher levels of phosphorylation of TH, we also find increased levels of phosphorylation of DARPP-32. In particular, neglecting dams have increased optical density for phosphorylated DARPP-32 (pDARPP-32) immunoreactivity within the nucleus accumbens and CP (Fig. 7). Nucleus accumbens is involved in maternal care [38], [39] and is an important component of the reward system [58]. D1-like receptor binding in nucleus accumbens is decreased in late pregnancy [59], suggesting some changes to D1-like receptor expression is linked to the onset of maternal care. While the CP appears to play a role in reward related processes [11], less is known about its functional role in regulating maternal care. DARPP-32 Thr-34 mutant mice lack the induction of △FosB, which typically accumulates within nucleus accumbens and dorsal striatum after repeated administration of drugs of abuse, and exhibit reduced reward response to cocaine [60]. It will be interesting to determine whether the dysregulated dopamine in neglectful MaD1 mice disrupts the rewarding properties of maternal-pup interactions, resulting in neglect of offspring.

Elevated pDARPP-32 levels with neglect are also of interest because progesterone can affect [61], suggesting the possibility that altered steroid signaling contributes to neglect. However, a hormonal profile of neglectful versus nurturing MaD1 mice during pregnancy and parturition has not been examined. Future work on this may provide important insights into possible hormonal contributions to neglect.

The altered c-Fos, pTH, and pDARPP-32 activity in neglectful mice is consistent with altered dopamine signaling. Elevated pTH in VTA (a key dopamine releasing region) in neglectful mice did not reach significance, but c-Fos did. It is possible that altered dopamine production occurs in VTA and that another approach will be needed to evaluate this. The differences seen in c-Fos expression compared to pTH and pDARPP-32 expression also indicate that dopamine may not be the only player influencing neglect.

Our findings implicating dopamine dysregulation in the production of naturally occurring neglect are consistent with a number of prior studies that suggest dopamine involvement in neglect. For example, disruption of D1, D2, or D4 dopamine receptors in medial preoptic area impairs differing aspects of maternal care in rats [15]. Lesions of VTA and nucleus accumbens disrupt maternal behaviors [16]–[19] and depletion of dopamine within ventral striatum specifically disrupts pup retrieval [62]. Elevating dopamine levels in rats prepartum (via cocaine) impairs maternal care [63], [64] and human mothers who used cocaine during pregnancy show decreased interaction with babies [65].

Normal maternal care involves, in part, linking response to offspring with natural reward systems. For example, lactating females will bar press for pups [16] and pup stimuli are more rewarding than cocaine for lactating rat dams using a conditioned place paradigm [66]. Further, recent work using fMRI scans in rats showed that in lactating dams, addiction and reward brain regions show much greater activation with pups than cocaine as a stimulus and that this effect is reversed in virgin females [37]. Thus, the production of normal maternal responding involves an alteration of natural reward pathways to respond to offspring as highly rewarding. Our results support previous studies that suggest dysregulated dopamine may reflect a lack of proper modification of reward pathways and may play a central role in child neglect. In each generation of MaD1 mice, we observe extreme neglect in a consistent proportion of the population and the background population from which MaD1 mice were derived may have altered dopamine signaling, for review, see [67]. However, most of the MaD1 mice are excellent mothers in all aspects, including protection of offspring [23], [24]. Therefore within this MaD1 population, some individuals, possibly at the high end of the spectrum of dopamine signaling, may be vulnerable to exhibit neglect as mothers. The MaD1 mice, then, provide an excellent model for examining how maternal neglect appears regularly within an otherwise normal, maternally nurturing population.

### Summary

The current study characterizes a population of mice (MaD1) in which an average of 17% naturally neglect their offspring per generation. We have identified a set of behavioral risk factors, such as decreased self-grooming and increased activity, which are associated with maternal neglect. As elucidated by the cross-fostering study, there are possible epigenetic contributions to neglect. That is, offspring raised by neglectful mothers will then exhibit decreased maternal care as adults, suggesting a mother-to-offspring transference of neglect. We also report that offspring born to previously neglectful dams can elicit heightened maternal neglect when cross-fostered to normally nurturing mothers, suggesting that factors regulating maternal neglect can be triggered by the offspring. While the biological basis for maternal neglect is not known, we found that atypical dopamine activity might be one factor regulating maternal neglect of offspring. In conclusion, these data suggest that MaD1 mice can be utilized as a powerful model for examining the biological basis of naturally occurring neglect, as well as the mother-offspring relationships that regulate neglect.

## Materials and Methods

### Animals

MaD1, Outbred-S, and outbred hsd:ICR (Harlan, Madison, WI) mice were used. MaD1 mice have been maintained in our lab for over 17 generations using selection for high maternal defense. Outbred-S mice were derived by us from outbred hsd:ICR mice also using selection for high maternal defense [25] and an on-going colony is maintained in the lab. For mating in these studies, females were housed with a single male of the same strain (unless noted). After two weeks of co-housing, males were removed and each female remained housed individually when raising offspring. Female mice were given ad lib access to Breeder Chow (Harlan) and tap water. Bedding was a combination of shaved aspen wood chips and Cellu-Dri (Shepherd Specialty Paper, Watertown, TN), both of which are used for nest building. All mice were housed on a 14:10 light/dark cycle with lights on at 0600 CST. All procedures followed the guidelines of the National Institutes of Health Guide for the Care and Use of Laboratory Animals, and were approved by the Animal Care and Use Committee of the University of Wisconsin.

### Evaluation of behavior prepartum, maternal neglect rate, and other maternal features

Prior to pairing with a male, MaD1 females were examined for general behaviors for Generations 6, 12, and 15. All observations were made of mice in their home cage in the home room and took place in the morning for 1 hour between 0900 and 1000. Observations included self-grooming and activity (locomotion, flipping or cage top climbing). In Generation 12, general behavioral observations of the females were also conducted during the second week of pregnancy. For all behavioral observations here and below, mice were coded and recordings were made by individuals blind to experimental conditions. Maternal neglect rate was determined by examining the number of live litters on postpartum Day 5 relative to the number of live litters born. Birth rate was determined as the number of live births relative to the number of females paired with males for breeding. Litter size was determined by counting live pups on postpartum Day 0. In Generation 16, first time mothers were examined 3 times a day (∼8–10 am; 4–6 pm; and 10–12 pm) from postpartum Day 0–7. Maternal features, such as nursing, and pup features, such as milk in stomach and pup death, were recorded. In Generation 17, pup retrieval was examined on postpartum Day 0 on mothers with their second litter.

Evaluation of maternal neglect rate and birth rate between MaD1 and Outbred-S mice was made using a one-way ANOVA with data from each of 12 generations for each group. For each generation, approximately 80 lactating females were examined per group. For examination of maternal neglect rate on a second litter a one-way ANOVA was used. For examining risk factors with neglect, if data were collected from multiple generations, then generation itself was included as a variable and a 2-way ANOVA was used. In cases here and below where the data were not normally distributed, either transformations were used to achieve normality before running the ANOVA or non-parametric tests were used.

### Light/dark box testing

Mice were placed in the dark portion of the light/dark box to initiate the 5 min test session. Time spent in the light and dark portions of the box were recorded with time in light portion of the box defined as entry of all four paws into this region. Mice were examined prior to mating both while they were group-housed and also following one week of individual housing. All behaviors were recorded on videotape and subsequently analyzed off-line. The light/dark box was used as a tool for examining levels of anxiety [68]–[70]. Results were analyzed using a one-way ANOVA between neglectful and nurturing MaD1 mice.

### Forced swim test

In Generation 17, at first sign of neglect (in almost all cases Day 1), neglectful and nurturing (stage matched) mice were placed in a glass cylinder (30 cm tall, 15 cm diameter) that was half filled with room temperature water and tested for 5 min. Time spent swimming and floating were recorded. Additionally, number of fecal boli produced while in the water were counted at the end of the session. All behaviors were recorded on videotape and subsequently analyzed off-line. Results were analyzed using a one-way ANOVA between neglectful and nurturing MaD1 mice.

### Cross-fostering of pups and maternal behavior examination

In order to examine the effects of maternal rearing environment and genotype on maternal neglect and offspring performance as adults, a cross-fostering study was conducted using mice on their second litter. Neglectful and nurturing MaD1 mice were identified based on performance with their first litter. Outbred-S mice that successfully raised pups for their first litter were also used. All mice were age matched and bred with mice of the same strain at the same time. At birth, all litters were cross-fostered among the three groups. Thus, 9 groups were created with ∼10 lactating mice per group. Previously neglectful MaD1 dams cared for pups from either a) previously neglectful MaD1 mice; b) previously nurturing MaD1 mice; or c) previously nurturing Outbred-S mice. Previously nurturing MaD1 and Outbred-S mothers also cared for these three groups. In all cases, the cross-fostering was completed whereby no dam raised her own pups. A maximum of 11 and minimum of 9 pups were cross-fostered per dam. On postpartum Day 3 of cross-fostering, behaviors of dams were examined in the home room for one hour. Behaviors were recorded every 30 sec using handheld Palm Pilots (Palm, Inc, Sunnyvale, CA) and information was downloaded to a computer for analysis. At age 21 days, pups were weaned and the percent pup survival was recorded. When cross-fostered pups were adults, behavioral measures were examined both prior to mating and during lactation. Cross-fostered female mice were mated as adults with an outbred (hsd:ICR strain) breeder male and examined for maternal behaviors. Maternal aggression and pup retrieval were examined using techniques previously described [25], [71], [72]. In brief, each lactating female was tested for 5 min for aggression using an outbred, group-housed intruder male (hsd:ICR) strain (never more than three tests per male). All tests were recorded on videotape and analyzed off-line by individuals blind to experimental conditions. On postpartum Day 10, features of pups were recorded, including pup weight and percent survival of pups (relative to total born). Examination of maternal behavior ceased after Day 10.

For analysis, a 2-way ANOVAs (using maternal rearing group and pup group) were used. Also, planned comparison one-way ANOVAs were used. For example, for examining the effect of rearing environment on offspring adult behavior, comparisons were made between common offspring that were raised by either neglectful versus nurturing MaD1 mothers.

### Immunohistochemistry for c-Fos, pTH, TH, and pDARPP-32

Beginning on postpartum Day 0 in Generation 16, all MaD1 mice were monitored for maternal behaviors twice per day. At first sign of maternal neglect, brains from neglectful mice were immediately collected. All brains were collected on Days 1 or 2 (the majority collected on Day 1), so this variable was not used as a covariate. Control brains from maternally normal mice were collected at the same time and the timing from birth to brain collection was identical for both groups. In Generation 17, previously neglectful or nurturing dams were sacrificed on postpartum day 0 of their second litter and processed for pDARPP-32.

For all brain collections, mice were decapitated following light isoflurane anesthesia and the brains removed. Brains were post-fixed overnight in 5% acrolein (Sigma) in phosphate buffered saline (PBS) and cryoprotected in 30% sucrose in PBS for two days. Brains were frozen on a platform and cut into 40 micron thick coronal sections using a sliding microtome (Leica, Microsystems, Heidelberg, Germany) and stored in a cryoprotectant solution at −20 degrees C until processing for immunohistochemistry. For each antibody used, immunohistochemistry was run for all mice in both groups in one batch.

For c-Fos, sections were incubated with 0.5% sodium borohydride for 30 min, washed in PBS in the presence of 0.2% Triton-X-100 (PBS-X), and blocked in 5% NGS for 1 hr. Sections were then incubated for two days at 4 degrees C in rabbit anti-c-Fos (1∶15,000; Calbiochem, La Jolla, CA; catalog # PC38). After washes in PBS-X, the sections were incubated for 90 min at room temperature with anti-rabbit secondary antibodies (1∶500, Vector), washed in PBS-X, exposed to an avidin-biotin complex (Vector) for 1 hr, washed again in PBS-X, and stained using diaminobenzidine (Sigma) enhanced with 0.008% nickel chloride. The sections were then mounted, dehydrated in a series of ethyl alcohols and xylenes, and coverslipped.

For pTH, TH and pDARPP-32, sections were washed three times in 0.1 M tris-buffered saline (TBS, pH 7.4) and then in TBS containing 0.1% sodium borohydride for 15 minutes. Following three more washes, sections were placed for 1 hour in TBS containing 20% normal goat serum (NGS) and 3% hydrogen peroxide. Tissue sections were then incubated overnight at room temperature in rabbit anti- ser40 pTH (1∶2,000; GeneTex, San Antonio, TX; catalog # GTX16557), mouse anti-TH (1∶5,000; GeneTex; catalog # GTX30172), or rabbit anti-pDARPP-32 (1∶1,000; Zymed, San Francisco, CA; catalog # 38-7500) in TBS containing 0.3% Triton X-100 (TBST), 2% NGS, and 0.5% gelatin. Following primary incubation, sections were washed three times in TBST and incubated in TBST containing the appropriate secondary antibody (1∶500, Vector Laboratories, Burlingame, CA) and 0.5% gelatin for ninety minutes. Following further washes, sections were exposed to an avidin-biotin complex (1∶400, Vector) for 1 hr, washed three times in TBST, and visualized using Vector SG (1∶167, Vector). Developed sections were mounted on gelatin-coated slides and coverslipped. Because the anti-TH antibodies were made in mouse, it was possible that the anti-mouse secondary would react with mouse brain tissue. However, when running control sections with just secondary anti-mouse antibodies, no staining occurred indicating that staining with primary antibodies was specific.

### Analysis of c-Fos, pTH, TH, and pDARPP-32 immunoreactivity

Bright field microscopy was used for counting c-Fos-positive cells. The images of brain sections were projected from an Axioskop Zeiss light microscope using a 10× objective (Zeiss, Gottingen, Germany) through an Axiocam Zeiss high resolution digital camera attached to the microscope and interfaced with a computer. Counting from specified brain regions was based on a previously used paradigm [43], [44], [73]. Brain regions were chosen for examination based on whether they had previously been implicated maternal care and/or dopamine signaling. For locations of most regions examined, see [43], [44], [74]. One section per brain region was used to quantify c-Fos immunoreactivity in each animal and a box of a preset size was used for a given region. The box was placed in the same site within a given region using overt landmarks. To ensure c-Fos was measured consistently between samples; 1) all sections were exposed to diaminobenzidine for 10 min, 2) the backgrounds were normalized by adjusting light levels, 3) a threshold of staining levels was used to automatically distinguish between c-Fos-positive cells, 4) all slides were coded and the counting for each specific brain region was performed by one individual, blind to the experimental conditions, and 5) only c-Fos-positive nuclei within a specified size range were counted. Evaluation of labeling for c-Fos between neglectful and nurturing MaD1 mice was conducted using a one-way ANOVA for each brain region. In the cases where the data were not normally distributed, then nonparametric tests were used.

Bilateral analyses of pTH, TH, and pDARPP-32 for one brain section per region were conducted using an Olympus BX61 microscope fitted with an Olympus FV II digital camera, connected to a PC compatible computer. The software used for analysis was Olympus MicroSuite (Soft Imaging System Corp., Lakewood, CO). Thresholds to detect foreground were set independently for each measurement to account for possible variability in background staining. The threshold was determined automatically by the imaging software, and was approximately 3× the standard deviation greater than the gray value mean of the background staining. Staining with a gray value greater than the threshold was detected and the total area covered by positive staining was obtained using the detection setting within the Olympus Microsuite software program. We have used this method of detection threshold of around 3X the standard deviation of mean background routinely with great success [75]–[77]. This method detects staining of cell bodies, fibers and punctate structures. The optical density of positive staining was also quantified on a 0 to 4095 scale of a 12-bit camera. After inverting the camera scale, a value of 0 corresponds to white and a value of 4095 to black. Evaluation of pTH, TH, and pDARPP-32 were analyzed using a one-way ANOVA for each brain region.

## References

[1] Maestripieri D, Carroll KA (1998). Child abuse and neglect: usefulness of the animal data.. Psychol Bull.

[2] Lounds JJ, Borkowski JG, Whitman TL (2004). Reliability and validity of the mother-child neglect scale.. Child Maltreat.

[3] U. S. Department of Health and Human Services Administration for Children and Families (2004). Child maltreatment 2002: summary of key findings.

[4] De Bellis MD (2005). The psychobiology of neglect.. Child Maltreat.

[5] Hildyard KL, Wolfe DA (2002). Child neglect: developmental issues and outcomes.. Child Abuse Negl.

[6] Lounds JJ, Borkowski JG, Whitman TL (2006). The potential for child neglect: the case of adolescent mothers and their children.. Child Maltreat.

[7] Shipman K, Edwards A, Brown A, Swisher L, Jennings E (2005). Managing emotion in a maltreating context: a pilot study examining child neglect.. Child Abuse Negl.

[8] Wilson SL, Kuebli JE, Hughes HM (2005). Patterns of maternal behavior among neglectful families: implications for research and intervention.. Child Abuse Negl.

[9] Lang AJ, Rodgers CS, Lebeck MM (2006). Associations between maternal childhood maltreatment and psychopathology and aggression during pregnancy and postpartum.. Child Abuse Negl.

[10] Numan M, Insel TR (2003). The neurobiology of parental behavior.

[11] Kelley AE, Berridge KC (2002). The neuroscience of natural rewards: relevance to addictive drugs.. J Neurosci.

[12] Byrnes EM, Rigero BA, Bridges RS (2002). Dopamine antagonists during parturition disrupt maternal care and the retention of maternal behavior in rats.. Pharmacol Biochem Behav.

[13] Numan M, Numan MJ, Pliakou N, Stolzenberg DS, Mullins OJ (2005). The effects of D1 or D2 dopamine receptor antagonism in the medial preoptic area, ventral pallidum, or nucleus accumbens on the maternal retrieval response and other aspects of maternal behavior in rats.. Behav Neurosci.

[14] Miller SM, Lonstein JS (2005). Dopamine d1 and d2 receptor antagonism in the preoptic area produces different effects on maternal behavior in lactating rats.. Behav Neurosci.

[15] Silva MRP, Bernardi MM, Felicio LF (2001). Effects of dopamine receptor antagonists on ongoing maternal behavior in rats.. Pharmacol Biochem Behav.

[16] Lee A, Clancy S, Fleming AS (2000). Mother rats bar-press for pups: effects of lesions of the mpoa and limbic sites on maternal behavior and operant responding for pup-reinforcement.. Behav Brain Res.

[17] Gaffori O, Lemoal M (1979). Disruption of maternal-behavior and appearance of cannibalism after ventral mesencephalic tegmentum lesions.. Physiol Behav.

[18] Numan M, Smith HG (1984). Maternal-behavior in rats - evidence for the involvement of preoptic projections to the ventral tegmental area.. Behav Neurosci.

[19] Hansen S, Harthon C, Wallin E, Lofberg L, Svensson K (1991). Mesotelencephalic dopamine system and reproductive behavior in the female rat: effects of ventral tegmental 6-hydroxydopamine lesions on maternal and sexual responsiveness.. Behav Neurosci.

[20] Francis DD, Young LJ, Meaney MJ, Insel TR (2002). Naturally occurring differences in maternal care are associated with the expression of oxytocin and vasopressin (V1a) receptors: gender differences.. J Neuroendocrinol.

[21] Champagne F, Meaney MJ (2001). Like mother, like daughter: evidence for non-genomic transmission of parental behavior and stress responsivity.. Prog Brain Res.

[22] Swallow JG, Carter PA, Garland T (1998). Artificial selection for increased wheel-running behavior in house mice.. Behav Genet.

[23] Girard I, Swallow JG, Carter PA, Koteja P, Rhodes JS (2002). Maternal-care behavior and life-history traits in house mice (*Mus domesticus*) artificially selected for high voluntary wheel-running activity.. Behav Process.

[24] Gammie SC, Hasen NS, Rhodes JS, Girard I, Garland T (2003). Predatory aggression, but not maternal or intermale aggression, is associated with high voluntary wheel-running behavior in mice.. Horm Behav.

[25] Gammie SC, Garland T, Stevenson SA (2006). Artificial selection for increased maternal defense behavior in mice.. Behav Genet.

[26] Brayden RM, Altemeier WA, Tucker DD, Dietrich MS, Vietze P (1992). Antecedents of child neglect in the 1st 2 years of life.. J Pediatr.

[27] Strathearn L, Gray PH, O'Callaghan MJ, Wood DO (2001). Childhood neglect and cognitive development in extremely low birth weight infants: A prospective study.. Pediatrics.

[28] Solanto MV (2002). Dopamine dysfunction in AD/HD: integrating clinical and basic neuroscience research.. Behav Brain Res.

[29] Murray C, Johnston C (2006). Parenting in mothers with and without attention-deficit/hyperactivity disorder.. J Abnorm Psychol.

[30] Cryan JF, Page ME, Lucki I (2005). Differential behavioral effects of the antidepressants reboxetine, fluoxetine, and moclobemide in a modified forced swim test following chronic treatment.. Psychopharmacology (Berl).

[31] Cryan JF, Mombereau C (2004). In search of a depressed mouse: utility of models for studying depression-related behavior in genetically modified mice.. Mol Psychiatry.

[32] Bale TL, Vale WW (2003). Increased depression-like behaviors in corticotropin-releasing factor receptor-2-deficient mice: sexually dichotomous responses.. J Neurosci.

[33] Prickaerts J, Moechars D, Cryns K, Lenaerts I, van Craenendonck H (2006). Transgenic mice overexpressing glycogen synthase kinase 3beta: a putative model of hyperactivity and mania.. J Neurosci.

[34] Abler B, Greenhouse I, Ongur D, Walter H, Heckers S (2007). Abnormal reward system activation in mania.. Neuropsychopharmacology.

[35] Fresiello A, Grammatikopoulos G, Pignatelli M, Sadile AG (2002). Environmental factors during postnatal period modify activity and non-selective attention in the Naples High-Excitability rat.. Behav Brain Res.

[36] Champagne FA, Weaver ICG, Diorio J, Dymov S, Szyf M (2006). Maternal care associated with methylation of the estrogen receptor-alpha 1b promoter and estrogen receptor-alpha expression in the medial preoptic area of female offspring.. Endocrinology.

[37] Ferris CF, Kulkarni P, Sullivan JM, Harder JA, Messenger TL (2005). Pup suckling is more rewarding than cocaine: evidence from functional magnetic resonance imaging and three-dimensional computational analysis.. J Neurosci.

[38] Numan M, Numan MJ, Schwarz JM, Neuner CM, Flood TF (2005). Medial preoptic area interactions with the nucleus accumbens-ventral pallidum circuit and maternal behavior in rats.. Behav Brain Res.

[39] Stolzenberg DS, McKenna JB, Keough S, Hancock R, Numan MJ (2007). Dopamine D1 receptor stimulation of the nucleus accumbens or the medial preoptic area promotes the onset of maternal behavior in pregnancy-terminated rats.. Behav Neurosci.

[40] Lavi-Avnon Y, Weller A, Finberg JP, Gispan-Herman I, Kinor N (2008). The reward system and maternal behavior in an animal model of depression: a microdialysis study.. Psychopharmacology (Berl).

[41] Afonso VM, Grella SL, Chatterjee D, Fleming AS (2008). Previous maternal experience affects accumbal dopaminergic responses to pup-stimuli.. Brain Res.

[42] Aragona BJ, Liu Y, Yu YJ, Curtis JT, Detwiler JM (2006). Nucleus accumbens dopamine differentially mediates the formation and maintenance of monogamous pair bonds.. Nat Neurosci.

[43] Gammie SC, Negron A, Newman SM, Rhodes JS (2004). Corticotropin-releasing factor inhibits maternal aggression in mice.. Behav Neurosci.

[44] D'Anna KD, Stevenson SA, Gammie SC (2005). Urocortin 1 and 3 impair maternal defense behavior in mice.. Behav Neurosci.

[45] Wagner CK, Eaton MJ, Moore KE, Lookingland KJ (1995). Efferent projections from the region of the medial zona incerta containing a(13) dopaminergic-neurons - a Pha-L anterograde tract-tracing study in the rat.. Brain Res.

[46] Kumer SC, Vrana KE (1996). Intricate regulation of tyrosine hydroxylase activity and gene expression.. J Neurochem.

[47] Dunkley PR, Bobrovskaya L, Graham ME, von Nagy-Felsobuki EI, Dickson PW (2004). Tyrosine hydroxylase phosphorylation: regulation and consequences.. J Neurochem.

[48] Zigmond RE, Schwarzschild MA, Rittenhouse AR (1989). Acute regulation of tyrosine hydroxylase by nerve activity and by neurotransmitters via phosphorylation.. Annu Rev Neurosci.

[49] Harada, Wu J, Haycock JW, Goldstein M (1996). Regulation of L-DOPA biosynthesis by site-specific phosphorylation of tyrosine hydroxylase in AtT-20 cells expressing wild-type and serine 40-substituted enzyme.. J Neurochem.

[50] Lindgren N, Goiny M, Herrera-Marschitz M, Haycock JW, Hokfelt T (2002). Activation of extracellular signal-regulated kinases 1 and 2 by depolarization stimulates tyrosine hydroxylase phosphorylation and dopamine synthesis in rat brain.. Eur J Neurosci.

[51] Lookingland KJ, Moore KE (1985). Acute effects of morphine on neurochemical estimates of activity of incertohypothalamic dopaminergic-neurons in the male-rat.. Brain Res.

[52] Sita LV, Elias CF, Bittencourt JC (2003). Dopamine and melanin-concentrating hormone neurons are distinct populations in the rat rostromedial zona incerta.. Brain Res.

[53] Ouimet CC, Miller PE, Hemmings HC, Walaas SI, Greengard P (1984). Darpp-32, a dopamine and adenosine 3′-5′-monophosphate-regulated phosphoprotein enriched in dopamine-innervated brain-regions .3. Immunocytochemical localization.. J Neurosci.

[54] Walaas SI, Greengard P (1984). Darpp-32, a dopamine and adenosine 3′-5′-monophosphate-regulated phosphoprotein enriched in dopamine-innervated brain-regions .1. Regional and cellular-distribution in the rat-brain.. J Neurosci.

[55] Hemmings HC, Nairn AC, Aswad DW, Greengard P (1984). Darpp-32, a dopamine and adenosine 3′-5′-monophosphate-regulated phosphoprotein enriched in dopamine-innervated brain-regions .2. Purification and characterization of the phosphoprotein from bovine caudate-nucleus.. J Neurosci.

[56] Langley KC, Bergson C, Greengard P, Ouimet CC (1997). Co-localization of the D-1 dopamine receptor in a subset of DARPP-32-containing neurons in rat caudate-putamen.. Neuroscience.

[57] Risinger FO, Freeman PA, Greengard P, Fienberg AA (2001). Motivational effects of ethanol in DARPP-32 knock-out mice.. J Neurosci.

[58] Carelli RM (2002). The nucleus accumbens and reward: neurophysiological investigations in behaving animals.. Behav Cogn Neurosci Rev.

[59] Bakowska JC, Morrell JI (1995). Quantitative autoradiographic analysis of D1 and D2 dopamine receptors in rat brain in early and late pregnancy.. Brain Res.

[60] Zachariou V, Sgambato-Faure V, Sasaki T, Svenningsson P, Berton O (2006). Phosphorylation of DARPP-32 at Threonine-34 is required for cocaine action.. Neuropsychopharmacology.

[61] Mani SK, Fienberg AA, O'Callaghan JP, Snyder GL, Allen PB (2000). Requirement for DARPP-32 in progesterone-facilitated sexual receptivity in female rats and mice.. Science.

[62] Hansen S, Harthon C, Wallin E, Lofberg L, Svensson K (1991). The effects of 6-OHDA-induced dopamine depletions in the ventral or dorsal striatum on maternal and sexual behavior in the female rat.. Pharmacol Biochem Be.

[63] Johns JM, Noonan LR, Zimmerman LI, Li L, Pedersen CA (1994). Effects of chronic and acute cocaine treatment on the onset of maternal behavior and aggression in Sprague-Dawley rats.. Behav Neurosci.

[64] Johns JM, Elliott DL, Hofler VE, Joyner PW, McMurray MS (2005). Cocaine treatment and prenatal environment interact to disrupt intergenerational maternal behavior in rats.. Behav Neurosci.

[65] Light KC, Grewen KM, Amico JA, Boccia M, Brownley KA (2004). Deficits in plasma oxytocin responses and increased negative affect, stress, and blood pressure in mothers with cocaine exposure during pregnancy.. Addict Behav.

[66] Mattson BJ, Williams S, Rosenblatt JS, Morrell JI (2001). Comparison of two positive reinforcing stimuli: pups and cocaine throughout the postpartum period.. Behav Neurosci.

[67] Rhodes JS, Gammie SC, Garland T (2005). Neurobiology of mice selected for high voluntary wheel-running activity.. Integr Comp Biol.

[68] Bouwknecht JA, Paylor R (2002). Behavioral and physiological mouse assays for anxiety: a survey in nine mouse strains.. Behav Brain Res.

[69] Gimenez-Llort L, Fernandez-Teruel A, Escorihuela RM, Fredholm BB, Tobena A (2002). Mice lacking the adenosine A1 receptor are anxious and aggressive, but are normal learners with reduced muscle strength and survival rate.. Eur J Neurosci.

[70] Henry B, Vale W, Markou A (2006). The effect of lateral septum corticotropin-releasing factor receptor 2 activation on anxiety is modulated by stress.. J Neurosci.

[71] D'Anna KD, Gammie SC (2006). Hypocretin-1 dose-dependently modulates maternal behaviour in mice.. J Neuroendocrinol.

[72] Gammie SC, Hasen NS, Stevenson SA, Bale TL, D'Anna KD (2005). Elevated stress sensitivity in corticotropin-releasing factor receptor 2 deficient mice decreases maternal, but not intermale aggression.. Behav Brain Res.

[73] Rhodes JS, Garland T, Gammie SC (2003). Patterns of brain activity associated with variation in voluntary wheel-running behavior.. Behav Neurosci.

[74] Hasen NS, Gammie SC (2005). Differential fos activation in virgin and lactating mice in response to an intruder.. Physiol Behav.

[75] Olesen KM, Jessen HM, Auger CJ, Auger AP (2005). Dopaminergic activation of estrogen receptors in neonatal brain alters progestin receptor expression and juvenile social play behavior.. Endocrinology.

[76] Olesen KM, Auger AP (2005). Sex differences in Fos protein expression in the neonatal rat brain.. J Neuroendocrinol.

[77] Olesen KM, Auger CJ, Auger AP (2007). Regulation of progestin receptor expression in the developing rat brain by a dopamine d1 receptor antagonist.. J Neuroendocrinol.

